# Factors associated with the length of hospital stay of women undergoing cesarean section

**DOI:** 10.11606/s1518-8787.2019053001113

**Published:** 2019-08-23

**Authors:** Samire Lopes Pereira, Thales Philipe Rodrigues da Silva, Alexandra Dias Moreira, Taiane Gonçalves Novaes, Milene Cristine Pessoa, Isabela Penido Matozinhos, Renato Camargo Couto, Tânia Moreira Grillo Pedrosa, Fernanda Penido Matozinhos

**Affiliations:** I Universidade Federal de Minas Gerais . Escola de Enfermagem . Curso de Especialização em Enfermagem Obstétrica – modalidade residência. Belo Horizonte , MG , Brasil; II Universidade Federal de Minas Gerais . Faculdade de Medicina . Programa de Pós-Graduação em Ciências da Saúde . Belo Horizonte , MG , Brasil; III Universidade Federal de Minas Gerais . Escola de Enfermagem . Departamento de Enfermagem Materno-Infantil e Saúde Pública . Belo Horizonte , MG , Brasil; IV Universidade Federal de Viçosa . Departamento de Nutrição . Programa de Pós-Graduação em Saúde e Nutrição de Grupos Populacionais . Viçosa , MG , Brasil; V Universidade Federal de Minas Gerais . Escola de Enfermagem . Departamento de Nutrição . Belo Horizonte , MG , Brasil; VI Faculdade de Ciências Médicas de Minas Gerais . Departamento de Medicina . Belo Horizonte , MG , Brasil; VII Instituto de Acreditação e Gestão em Saúde (IAG Saúde). Belo Horizonte , MG , Brasil

**Keywords:** Cesarean Section, rehabilitation, Hospitalization, Length of Stay, Comorbidity, Risk Factors

## Abstract

**OBJECTIVE:**

To evaluate whether age group, complications or comorbidities are associated with the length of hospitalization of women undergoing cesarean section.

**METHODS:**

A cross-sectional study was carried out between June 2012 and July 2017, with 64,437 women undergoing cesarean section and who did not acquire conditions during their hospital stay. Hospital discharge data were collected from national health institutions, using the Diagnosis-Related Groups system (DRG Brasil®). The DRG referring to cesarean section with additional complications or comorbidities (DRG 765) and cesarean section without complications or associated comorbidities (DRG 766) were included in the initial diagnosis. The influence of age group and comorbidities or complications present at admission on the length of hospital stay was assessed based on the means of the analysis of variance. The size of the effect was verified by Cohen’s D, which allows evaluating clinical relevance. The criticality levels were identified using the Duncan test.

**RESULTS:**

The longest length of hospital stay was observed in the age group from 15 to 17 years old and among those aged 45 years old or more. The hospital stay of women with complications or comorbidities at the time of admission was also longer. Moreover, it was noted that the increase in criticality level was associated with an increase in the mean length of hospital stay.

**CONCLUSIONS:**

The length of hospital stay of women is higher among those belonging to the age group ranging from 15 to 17 years old and for those aged 45 years old or more. The presence of associated comorbidities, such as eclampsia, pre-existing hypertensive disorder with superimposed proteinuria and gestational hypertension (induced by pregnancy) with significant proteinuria increase the length of hospital stay. This study enabled the construction of distinct criticality level profiles based on the combination of age groups and the main comorbidities, which were directly related to the length of hospital stay.

## INTRODUCTION

The prevalence of cesarean section has shown an increase in several countries of the world in the last decades ^1^ . In Brazil, in 1970, the cesarean section rate was about 15%, in 2001 it increased to 38% and, in 2008, to 48.8%, representing 35% of the childbirths in the Unified Health System (SUS) and 80% of those in the private sector. In 2009, the number of cesarean sections exceeded that of vaginal deliveries, representing 50.1% and, in 2012, 55.7% ^2^ of all deliveries. It should be noted that the World Health Organization (WHO), recommends a percentage between 10% and 15% as an acceptable cesarean section rate ^3^ .

However, considering the Brazilian population’s profile, in 2016, the Ministry of Health approved Ordinance 306, which indicates the need to adjust this rate to individual factors, such as the women’s demographic, clinical and obstetric characteristics (parity and previous caesarean section, among others), and structuring characteristics, such as the obstetric care model. In this way, the aforementioned rate is between 25% and 30% in the country ^4^ .

Evidence shows that, when performed unnecessarily, surgical deliveries may lead to a higher risk of puerperal infection, prematurity and neonatal mortality, in addition to increasing the length of hospital stay ^3 , 5^ . On the other hand, a decrease in the rates of maternal and neonatal mortality related to cesarean delivery may be noted in specific situations, such as in the presence of placenta previa or uterine rupture, making this type of delivery the safest one ^6^ . Therefore, cesarean sections may have positive health impacts when properly recommended, such as to reduce the bleeding index ^2^ .

The probability of the performance of cesarean section has been associated with factors pertaining to the mother, to the child and to pregnancy, without being limited to them. Maternal factors may include age, pre-existing comorbidities, previous cesarean section and health conditions acquired during pregnancy, among others. Those related to the child include problems identified in the intrapartum period, such as fetal anomalies, macrosomia and restricted intrauterine growth. Obstetric factors refer to conditions related to the current pregnancy, such as premature placental displacement, cord prolapse and hemorrhages ^7^ .

Considering that the length of hospital stay of patients for similar reasons is heterogeneous, that is, there is a coefficient of variation due to the differences in the care offered by the various health institutions and to the diversity in the patients’ individual characteristics, especially their complications and comorbidities, the objective of the study was to evaluate the impact of these clinical conditions and age group on the length of hospitalization of women undergoing cesarean section.

The determination of the influence of factors that are intrinsic to the patient on the length of hospital stay provides the opportunity to implement safe care management mechanisms, based on indicators of clinical risk adjustment to the length of hospitalization for cesarean section.

## METHODS

This is a cross-sectional study, carried out with 64,437 women aged between 15 and 59 years old undergoing cesarean section, from June 2012 to July 2017. All puerperal women who had been discharged to their homes and who did not acquire any conditions during the hospitalization period were considered. Acquired conditions refer to adverse events related to care and which are not directly determined by the patient’s clinical conditions.

Hospital discharge data were collected using the Diagnosis-Related Groups system (DRG Brasil®), version 9.04, from national health institutions that treat acute cases, that is, those where the average stay does not exceed 30 days ^8 , 9^ .

The DRG methodology is a classification system that takes into account the main diagnosis, comorbidities and complications present at admission, procedures performed, age and other variables of the patient. Clinically and economically homogeneous patients are grouped in each DRG. In other words, patients assigned to the same DRG share both clinical similarity and similarity of consumption of institutional resources, including length of stay. The DRG are organized according to physiological system in large diagnostic groups called MDC (Major Diagnostic Categories) ^10 , 11^ .

The design and development of the DRG began at the end of the 1960s at the Yale University in the United States of America (USA). It was originally devised with the purpose of subsiding hospital management, making it possible to measure and evaluate the performance of hospitals ^9^ .

The DRG methodology used in the USA was adapted to Brazil in the beginning of the 2000s by a group of researchers of the Lucas Machado Educational Foundation’s (Feluma) graduate course, based on the algorithms of the Medicare Severity DRG (MS-DRG). This group of researchers developed the DRG ® software, which has been in use in the country since 2011.

The database is generated from the reading of medical records by nurse encoders. The quality of the data is continually audited by physicians and nurses who specialize in the DRG Brasil® platform, reporting potential errors to the coding teams for revaluation.

The current database of DRG Brasil® covers approximately 400 Brazilian hospitals (72% with supplementary health care and 28% with public health care), distributed according to the country’s regions: 40.1% in the Southeast, 35.7% in the Midwest, 17.6% in the South, 5.5% in the North, and 1.1% in the Northeast. Around 1 million and 500 thousand hospital discharge records have been encoded. All clinical diagnoses, both primary and secondary, including complications and comorbidities, are classified according to the International Classification of Diseases (ICD), tenth revision, eighth edition.

This study included the DRG referring to cesarean section with additional complications or comorbidities (DRG 765) and cesarean section without complications or associated comorbidities (DRG 766) that are part of MDC14 (pregnancy, childbirth and puerperium). Absolute and relative frequencies were used to present the descriptive data. The behavior of the length of stay, in day(s), was evaluated based on the mean, standard deviation (SD) and percentiles of interest (P10, P25, P50, P75 and P90). An analysis of variance (ANOVA) was performed to evaluate the effect of age group and comorbidities or complications at the time of admission on the length of hospital stay of women undergoing cesarean section. The length of hospital stay in days of hospitalization was considered as dependent variable, whereas age group (15 to 17, 18 to 24, 25 to 34, 35 to 44 and 45 years old or more) and the complications and comorbidities at the time of admission of at least 40 individuals were considered as independent variables, a total of 81 complications or comorbidities having been evaluated.

Because this study used large samples, the effect size of the significant differences was verified, using Cohen’s D to assess significance, since there may be an increase in the probability of type I error. Thus, the analysis of the effect’s size allows evaluating clinical relevance. It was classified as small (Cohen’s d < 0.40), moderate (Cohen’s D between 0.40 and 0.60) and large (Cohen’s ≥ 0.60). The factors influencing the length of hospital stay were those with a significant effect (P < 0.05) in ANOVA, and an effect size equal to or greater than 0.40.

It should be noted that many variables with statistical and practical relevance were identified. The existence of a true significant association between the ICD was verified with the clinical evaluation of each ICD, those that had no isolated clinical significance or that overlapped with other ICD for which Cohen’s d ≥ 0.60 having been disregarded.

After the identification of the factors indicated by factorial ANOVA and Cohen’s D, all possible profiles were constructed from the combination of factors with statistical and practical significance. Multiple comparisons of means were made according to the Duncan test or Fisher’s LSD, to verify between which profiles there really were differences. The objective of this analysis was to compare the means between three or more groups in relation to an interval variable (continuous or discrete) of interest to identify possible criticality levels. The requirements for the use of this analysis, the normality of the model’s residues and the presence of variance were verified.

The project was approved by the Research Ethics Committee of the Federal University of Minas Gerais’ School of Medicine, under opinion No. 34133814.5.0000.5149. The participants were asked to sign an informed consent form.

## RESULTS

Longer hospital stay of women belonging to the age group ranging from 15 to 17 years old and among those aged 45 years old or more was observed when compared to the other groups (18 to 24 years old, 25 to 34 years old and 35 to 44 years old), with statistically significant differences ( Table 1 ).

**Table 1:** Length of hospital stay of women undergoing cesarean section in relation to the age group of the diagnostic categories related to MDC14. Brazil, 2012–2017.

Variable	Hospital stay after caesarean section (days)		

	n (%)	Mean (SD)	p
Age group (years)			**0.050**
15 to 17 ^a^	632 (1.0)	2.5 (1.5)	
18 to 24 ^b^	8,004 (12.4)	2.3 (1.4)	
25 to 34 ^b^	37,498 (58.1)	2.2 (1.4)	
35 to 44 ^b^	18,100 (28.1)	2.3 (1.4)	
45 or more ^a^	239 (0.4)	2.6 (1.7)	

p-value in bold < 0.05 in factorial Anova.

Equal letters mean similarity between the groups’ means.

In relation to the ICD, of a total of 81, significant influence was identified for 63 of them. However, 34 of these ICD had a small effect (Cohen’s < 0.40) – that is, although there was statistical significance, there was no practical significance. Therefore, 29 ICD were individually evaluated, 12 of which showed large relevance (Cohen’d ≥ 0.60) and the others moderate relevance (Cohen’s between 0.40 and 0.60), as may be seen in Table 2 . The length of hospital stay of women undergoing cesarean section with complications or comorbidities was higher than that of women undergoing cesarean section without complications or comorbidities for all ICD.

**Table 2:** Length of hospital stay of women undergoing cesarean section in relation to complications or comorbidities present at admission according to the ICD of conditions related to MDC14. Brazil, 2012–2017.

Variable		Hospital stay after caesarean section (days)			

		Secondary comorbidities		Anova	Cohen’s D

ICD	ICD’s description	Absent Mean (SD)	Present Mean (SD)		
O15	Eclampsia	2.3 (1.4)	4.5 (2.2)	< 0.001	1.590
O11	Pre-existing hypertensive disorder with superimposed proteinuria	2.3 (1.4)	4.4 (2.0)	< 0.001	1.503
O14	Gestational hypertension (induced by pregnancy) with significant proteinuria	2.2 (1.4)	3.8 (2.0)	< 0.001	1.109
R60	Edema, not elsewhere classified	2.3 (1.4)	3.7 (2.1)	0.008	1.035
E14	Unspecified diabetes *mellitus*	2.3 (1.4)	3.5 (1.8)	< 0.001	0.840
O12	Gestational edema and proteinuria (induced by pregnancy), without hypertension	2.3 (1.4)	3.4 (1.6)	< 0.001	0.790
D69	Purpura and other haemorrhagic affections	2.3 (1.4)	3.2 (1.7)	0.010	0.686
D64	Other anemias	2.3 (1.4)	3.2 (2.0)	< 0.001	0.683
O26	Maternal care for other complications predominantly linked to pregnancy	2.3 (1.4)	3.2 (1.7)	< 0.001	0.619
E10	Insulin-dependent diabetes *mellitus*	2.3 (1.4)	3.1 (2.1)	0.004	0.585
O23	Genitourinary tract infections in pregnancy	2.3 (1.4)	3.1 (1.9)	< 0.001	0.577
O43	Placenta disorders	2.3 (1.4)	3.1 (2.0)	< 0.001	0.571
O30	Multiple pregnancies	2.3 (1.4)	3.0 (1.8)	< 0.001	0.537
O44	Placenta praevia	2.3 (1.4)	3.0 (1.8)	< 0.001	0.527
O60	Preterm labor	2.3 (1.4)	3.0 (1.8)	< 0.001	0.519
O84	Multiple childbirth	2.3 (1.4)	3.0 (1.7)	< 0.001	0.497

ICD: International Classification of Diseases

p-value in bold < 0.05 in Anova.

By combining age group and the 16 ICD with statistical and practical relevance, four criticality levels were identified among the women undergoing cesarean section. Criticality level 1 was composed of women undergoing cesarean section, independently of age group and without identification of complications or comorbidities at the time of admission. Criticality level 2 was composed of women from any age group undergoing cesarean section and with only one complication or comorbidity at the time of admission, which did not relate to ICD O15, O11, O14, E14 and O12, or the combination of ICD O60 and 84 (Table). Criticality level 3 consisted of women in the age group ranging from 18 to 44 years old, who had a combination of two or three complications or comorbidities at the time of admission, or ICD O11, O12, O14 and E14 only. Criticality level 4 consisted of women in the age group ranging from 15 to 17 years old and aged 45 years old or more, who had a combination of two or three complications or comorbidities at the time of admission, or ICD O11, O12, O14 and E14 only. It also included women from any age group with ICD O14 (gestational hypertension induced by pregnancy with significant proteinuria) combined with any other ICD with statistical and practical relevance other than ICD O15 (eclampsia). The women diagnosed with eclampsia were classified, independently of age group, as criticality level 4 (Table).

Finally, in Table 3 , the length of hospital stay according to the groups’ criticality level is shown. It may be noted that the mean length of stay increases with criticality level.

**Table 3:** Length of hospital stay of women undergoing cesarean section in relation to the criticality levels of the diagnostic categories related to MDC14. Brazil, 2012–2017.

Variable	Hospital stay after caesarean section (days)					

	Mean (SD)	p10	p25	p50	p75	p90
Level of criticality						
1	2.2 (1.3)	1.7	1.9	2.1	2.4	3.0
2	2.7 (1.7)	1.8	2.0	2.3	3.1	5.1
3	3.6 (1.9)	1.9	2.2	3.0	5.1	9.5
4	4.5 (2.0)	2.1	2.6	4.4	6.9	10.7

**Box:** Length of the criticality for hospital stay of women undergoing cesarean section of the diagnostic categories related to MDC14. Brazil, 2012–2017.

Level of criticality	Age group (years)	ICD															

		O15	O11	O14	R60	E14	O12	D69	D64	O26	E10	O23	O43	O30	O44	O60	O84
1	All	Absence of complications or comorbidities present at admission															
2	All	Presence of at least one complication or comorbidity present at admission (ICD O15, O11 and O14)															
3	18 to 44													Yes			Yes
	18 to 44													Yes		Yes	
	18 to 44													Yes		Yes	Yes
	18 to 44						Yes										
	18 to 44					Yes											
	18 to 44			Yes													
	18 to 44			Yes												Yes	
	18 to 44			Yes										Yes			
	18 to 44			Yes								Yes					
	18 to 44		Yes														
4	≤ 17 / ≥ 45													Yes			Yes
	≤ 17 / ≥ 45													Yes		Yes	
	≤ 17 / ≥ 45													Yes		Yes	Yes
	≤ 17 / ≥ 45						Yes										
	≤ 17 / ≥ 45					Yes											
	≤ 17 / ≥ 45			Yes													
	≤ 17 / ≥ 45			Yes												Yes	
	≤ 17 / ≥ 45			Yes										Yes			
	≤ 17 / ≥ 45			Yes								Yes					
	≤ 17 / ≥ 45		Yes														
	All											Yes				Yes	
	All			Yes													Yes
	All			Yes						Yes							
	All			Yes					Yes								
	All			Yes				Yes									
	All			Yes			Yes										
	All			Yes	Yes												
	All	Yes															

ICD: International Classification of Diseases

## DISCUSSION

This study showed that the hospital stay after cesarean section was longer for young (15 to 17 years old) and older women (45 years old or more) in relation to other age ranges. It was revealed that the presence of associated comorbidities, such as eclampsia, pre-existing hypertensive disorder with superimposed proteinuria and gestational hypertension (induced by pregnancy) with significant proteinuria, among others, increase the length of hospital stay. In addition, this study enabled the construction of distinct criticality level profiles based on the combination of age groups and the main comorbidities, which were directly related to the length of hospital stay.

A study conducted in 30 low and mid-income countries showed that the mean hospitalization after cesarean section ranged from 2.5 to 9.3 days in the studied localities. According to the findings of the present study, one of the factors associated with longer length of stay after the procedure was higher maternal age, as in a study conducted in the United States, in which more hospitalization days were associated with lower or higher age ranges (< 18 or > 35 years old) and multiple gestational comorbidities ^12^ . According to the authors, knowing the risk factors associated with prolonged hospitalization periods enables the realization of monitoring and surveillance actions during pregnancy, especially in the presence of more than one pathology ^13^ .

In Brazil, the number of women with late pregnancies has increased in the last years ^14^ . This occurs for several reasons, such as greater dedication to study and profession, high availability of contraceptive methods, as well as late marriage and choice of ideal partners ^15^ . However, high maternal age is often associated with gestational risks. A retrospective cohort study conducted in Texas with 96,879 participants showed higher rates of preeclampsia, eclampsia and placenta previa in pregnant women aged 40 years or more compared to those in the age group from 35 to 39 years old ^15^ . As in this study, the criticality levels of these women were higher, which should be considered in the planning of postpartum hospital stay and care, to ensure better perinatal outcomes.

The results of this study showed that hypertensive disorders during pregnancy are the comorbidities associated with prolonged hospitalization periods after cesarean section. These data are confirmed by previous studies, in which the presence of these complications was associated with higher cesarean section rates, prolonged hospitalization periods and hospital readmission, as well as high costs for the health system ^15 , 16^ . Thus, it emphasizes the importance of prevention and health promotion actions during prenatal care to minimize the burden of maternal morbidity and mortality in the population.

The present study also allowed classifying the women into four different criticality levels and demonstrated the need to prolong the length of hospitalization from the association between the sum of comorbidities in pregnancy and maternal age, considering diverse clinical needs. The recognition of the different obstetric risks and criticality levels of women undergoing cesarean section contributes to the development of strategies to minimize the length of hospital stay and subside decision-making in clinical practice. In this sense, the present study supports the creation of cutoff points from the stratification of these women so they may receive adequate and safe care based on an estimate of the expected hospitalization time.

A limitation of these findings is the non-representativeness of the sample, since the hospitals were not chosen at random. The database available to researchers was used, a convenience sample that may not represent the totality of the Brazilian hospital network. However, the potential of the risk frameworks presented here to subsidize the actions of managers and health professionals for the planning of the care of pregnant and postpartum women throughout the country is worth highlighting.

The DRG system is associated with the reduction in hospitalization time after caesarean section and other obstetric and gynecological procedures, in addition to reducing the rates of surgical deliveries ^17^ . This methodology enables the optimization of the use of hospital resources allied to the improvement in the quality of care and patient safety, directly impacting the reduction of maternal morbidity and mortality rates ^18^ .

## FINAL CONSIDERATIONS

The study showed that the DRG is a tool that influences the management of costs and care. With it, it was possible to define criticality levels, which allowed predicting the average length of hospital stay of women undergoing cesarean section. If the length of stay was greater than that calculated by the method, it would be indicative of a failure in the care process that, consequently, could increase hospital costs and generate burden for the patient.

The assessment of the impact of clinical conditions and age group on the length of hospital stay of women undergoing cesarean section is undoubtedly a factor to be considered when discussing the quality and efficiency of care. This study is therefore of importance for the planning of care and hospital management.
