# Supplementary material for: Factors associated with the length of hospital stay of women undergoing cesarean section
Source: Rev Saude Publica. 2019 Aug 23;53:65. doi: 10.11606/s1518-8787.2019053001113 (PMC6707620; doi:10.11606/s1518-8787.2019053001113)
Supplement: Translation

# Translation: Fatores associados ao tempo de permanência hospitalar de mulheres submetidas à cesariana

**Authors:** Samire Lopes Pereira, Thales Philipe Rodrigues da Silva, Alexandra Dias Moreira, Taiane Gonçalves Novaes, Milene Cristine Pessoa, Isabela Penido Matozinhos, Renato Camargo Couto, Tânia Moreira Grillo Pedrosa, Fernanda Penido Matozinhos

**Affiliations:** I Universidade Federal de Minas Gerais. Escola de Enfermagem. Curso de Especialização em Enfermagem Obstétrica – modalidade residência. Belo Horizonte, MG, Brasil; II Universidade Federal de Minas Gerais. Faculdade de Medicina. Programa de Pós-Graduação em Ciências da Saúde. Belo Horizonte, MG, Brasil; III Universidade Federal de Minas Gerais. Escola de Enfermagem. Departamento de Enfermagem Materno-Infantil e Saúde Pública. Belo Horizonte, MG, Brasil; IV Universidade Federal de Viçosa. Departamento de Nutrição. Programa de Pós-Graduação em Saúde e Nutrição de Grupos Populacionais. Viçosa, MG, Brasil; V Universidade Federal de Minas Gerais. Escola de Enfermagem. Departamento de Nutrição. Belo Horizonte, MG, Brasil; VI Faculdade de Ciências Médicas de Minas Gerais. Departamento de Medicina. Belo Horizonte, MG, Brasil; VII Instituto de Acreditação e Gestão em Saúde (IAG Saúde). Belo Horizonte, MG, Brasil

**Keywords:** Cesárea, reabilitação, Hospitalização, Tempo de Internação, Comorbidade, Fatores de Risco

## Abstract

**OBJETIVO:**

Avaliar se grupo etário, complicações ou comorbidades estão associados ao tempo de internação de mulheres submetidas à cesariana.

**MÉTODOS:**

Estudo transversal realizado entre junho de 2012 e julho de 2017, com 64.437 mulheres submetidas à cesariana e que não apresentaram condições adquiridas durante o tempo de permanência hospitalar. Os dados foram coletados a partir da alta hospitalar nas instituições nacionais de saúde, utilizando o sistema *Diagnosis-Related Groups* [Grupos de Diagnósticos Relacionados] (DRG Brasil®). Foram incluídos os DRG referentes à cesariana com complicações ou comorbidades adicionais ao diagnóstico inicial (DRG 765) e cesariana sem complicações ou comorbidades associadas (DRG 766). A influência do grupo etário e comorbidades ou complicações presentes na admissão sobre o tempo de permanência hospitalar foi avaliada por meio da análise de variância. O tamanho do efeito foi verificado pelo d de Cohen, que permite avaliar a relevância clínica. Os níveis de criticidade foram identificados utilizando o teste de Duncan.

**RESULTADOS:**

O maior tempo de permanência hospitalar foi observado nos grupos etários de 15 a 17 anos e 45 anos ou mais. Mulheres que apresentaram complicações ou comorbidades presentes à admissão também apresentaram maior tempo de permanência hospitalar. Quantos aos níveis de criticidade, notou-se que seu aumento estava associado ao aumento na média do tempo de permanência hospitalar.

**CONCLUSÕES:**

O tempo de permanência hospitalar de mulheres é maior entre aquelas pertencentes aos grupos etários de 15 a 17 anos e 45 anos ou mais. A presença de comorbidades associadas, como a eclâmpsia, o distúrbio hipertensivo pré-existente com proteinúria superposta e a hipertensão gestacional (induzida pela gravidez) com proteinúria significativa aumentam o tempo de permanência hospitalar. Este estudo possibilitou a construção de perfis distintos de níveis de criticidade a partir da combinação de grupos etários e das principais comorbidades, os quais se apresentaram diretamente relacionados ao tempo de permanência hospitalar.

## INTRODUÇÃO

A prevalência da operação cesariana tem apresentado aumento em vários países do mundo nas últimas décadas^1^ . No Brasil, em 1970, a taxa de cesáreas era de cerca de 15%, em 2001 passou para 38% e, em 2008, para 48,8%, representando 35% das vias de nascimento do Sistema Único de Saúde (SUS) e 80% do setor privado. Em 2009, o número de cesáreas ultrapassou o de partos vaginais, representando 50,1% e, em 2012, 55,7%^2^ de todos os partos. Ressalta-se que a Organização Mundial de Saúde (OMS), desde 1985, preconiza como taxa aceitável de cesárea um percentual entre 10% e 15%^3^ .

Contudo, considerando o perfil da população brasileira, em 2016 o Ministério da Saúde aprovou a Portaria 306, que aponta para a necessidade de ajuste dessa taxa em decorrência de fatores individuais, tais como características demográficas, clínicas e obstétricas das mulheres (paridade e cesariana prévia, entre outras), e estruturantes, como o modelo de atenção obstétrica. Dessa forma, atualmente a referida taxa situa-se entre 25% e 30% no país^4^ .

Evidências mostram que, quando realizados desnecessariamente, os partos cirúrgicos podem levar a um maior risco de infecção puerperal, prematuridade e mortalidade neonatal, além de elevarem o tempo de internação hospitalar^3 , 5^ . Por outro lado, observa-se diminuição das taxas de mortalidade materna e neonatal pelo parto cesariano em situações específicas, tais como na presença de placenta prévia ou de ruptura uterina, as quais tornam essa via de parto a mais segura^6^ . Pondera-se, portanto, que a cesariana pode apresentar impactos positivos para a saúde quando bem recomendada, como para reduzir o índice de hemorragias^2^ .

A probabilidade da realização de cesariana tem sido relacionada a fatores maternos, infantis e obstétricos, mas sem limitar-se a eles. Fatores maternos podem incluir idade, comorbidades pré-existentes, cesariana prévia e condições de saúde adquiridas durante a gravidez, entre outros. Fatores infantis incluem problemas identificados no período intraparto, como anomalias fetais, macrossomias e crescimento intrauterino restrito. Já os fatores obstétricos referem-se às condições relacionadas à gravidez atual, como deslocamento prematuro de placenta, prolapso de cordão e hemorragias^7^ .

Considerando que o tempo de permanência hospitalar das pacientes por motivo similar é heterogêneo, ou seja, há um coeficiente de variação devido à diferença na assistência nas diversas instituições de saúde e à diversidade nas características individuais das pacientes, principalmente das complicações e comorbidades presentes, o objetivo do estudo foi avaliar o impacto dessas condições clínicas e do grupo etário no tempo de internação das mulheres submetidas à cesariana.

A determinação da influência desses fatores intrínsecos à paciente no tempo de permanência hospitalar proporciona a oportunidade de se implantar mecanismos de gestão assistencial segura, a partir de referenciais de ajuste de risco clínico para tempo de internação hospitalar em cesariana.

## MÉTODOS

Trata-se de um estudo com delineamento transversal, realizado com 64.437 mulheres com idade entre 15 e 59 anos submetidas à cesariana, no período de junho de 2012 a julho de 2017. Foram consideradas todas as puérperas que tiveram alta para o domicílio e não apresentaram condições adquiridas durante o período de internação. Condições adquiridas referem-se a eventos adversos relacionados à assistência e que não são diretamente determinados pelas condições clínicas do paciente.

Os dados utilizados foram coletados utilizando o sistema *Diagnosis-Related Groups* [Grupos de Diagnósticos Relacionados] (DRG Brasil®), versão 9.04, a partir das altas hospitalares de instituições nacionais de saúde que atendem casos agudos, ou seja, aquelas em que a permanência média não ultrapassa os 30 dias^8 , 9^ .

A metodologia DRG é um sistema de classificação que leva em consideração o diagnóstico principal, comorbidades e complicações presentes à admissão, procedimentos realizados, idade e outras variáveis do paciente. Em cada DRG são agrupados pacientes clinicamente e economicamente homogêneos. Ou seja, os pacientes atribuídos a um mesmo DRG compartilham tanto similaridade clínica quanto de consumo de recursos institucionais, incluindo o tempo de permanência. Os DRG são organizados de acordo com o sistema fisiológico em grandes grupos diagnósticos denominados MDC ( *Major Diagnostic Categories* )^10 , 11^ .

O desenho e o desenvolvimento do DRG começaram no final dos anos 1960 na Universidade de Yale, nos Estados Unidos da América (EUA). Foi originalmente construído com o intuito de instrumentalizar a gestão hospitalar, viabilizando a mensuração e avaliação do desempenho dos hospitais^9^ .

A metodologia DRG utilizada nos EUA foi adaptada ao Brasil no início dos anos 2000, por um grupo de pesquisadores vinculados à pós-graduação da Fundação Educacional Lucas Machado (Feluma), a partir dos algoritmos para a classificação do *Medicare Severity DRG* (MS-DRG). Este grupo de pesquisadores desenvolveu o software DRG Brasil^®^, que está em uso no país desde 2011.

A base de dados é gerada a partir de leitura de prontuários realizada por enfermeiros codificadores. A qualidade dos dados é continuamente auditada por médicos e enfermeiros especialistas do suporte do DRG Brasil®, com retorno dos erros potenciais às equipes codificadoras para reavaliação.

A base de dados atual do DRG Brasil® abrange aproximadamente 400 hospitais brasileiros (72% com atendimento à saúde suplementar e 28% à saúde pública), assim distribuídos de acordo com a região do país: 40,1% no Sudeste, 35,7% no Centro-Oeste, 17,6% no Sul, 5,5% no Norte e 1,1% no Nordeste. São em torno de 1 milhão e 500 mil registros de altas hospitalares codificados. Todos os diagnósticos clínicos, tanto o principal quanto os secundários, incluindo as complicações e comorbidades, são classificados segundo a Classificação Internacional de Doenças (CID), décima revisão, oitava edição.

Neste estudo, foram incluídos os DRG referentes à cesariana com complicações ou comorbidades adicionais ao diagnóstico inicial (DRG 765) e cesariana sem complicações ou comorbidades associadas (DRG 766), que fazem parte do MDC14 (gestação, parto e puerpério). Os dados descritivos foram apresentados por frequências absolutas e relativas. O comportamento do tempo de permanência, em dia(s), foi avaliado por meio da média, desvio-padrão (DP) e percentis de interesse (p10, p25, p50, p75 e p90). Para avaliar o efeito do grupo etário e das comorbidades ou complicações presentes à admissão sobre o tempo de permanência hospitalar em mulheres submetidas à cesariana, utilizou-se a metodologia da análise de variância (ANOVA). Considerou-se, como variável dependente, o tempo de permanência hospitalar em dias de internação; como variável independente, o grupo etário (15 a 17, 18 a 24, 25 a 34, 35 a 44 e 45 anos ou mais) e as complicações e comorbidades presentes à admissão em pelo menos 40 indivíduos, avaliando um total de 81 complicações ou comorbidades.

Por se tratar de um estudo em grandes amostras, foi verificado o tamanho de efeito ( *effect size* ) das diferenças significativas, utilizando o d de Cohen para avaliar a significância, uma vez que pode ocorrer aumento da probabilidade do erro tipo I. Assim, a análise do tamanho do efeito permite avaliar a relevância clínica. Ele foi classificado em pequeno (d de Cohen < 0,40), moderado (d de Cohen entre 0,40 e 0,60) e grande (d de Cohen ≥ 0,60). Foram considerados fatores que influenciam no tempo de permanência hospitalar aqueles que tiveram efeito significativo (p < 0,05) na ANOVA e com tamanho de efeito igual ou superior a 0,40.

Ressalta-se que foram identificadas muitas variáveis com relevância estatística e prática. A existência de verdadeira associação significativa entre os CID foi verificada por avaliação clínica de cada CID, sendo desconsiderados aqueles que não tinham significado clínico isolado ou que se sobrepunham a outros CID já contemplados com d de Cohen ≥ 0,60.

Após a identificação dos fatores evidenciados pela ANOVA fatorial e d de Cohen, foram construídos todos os perfis possíveis a partir da combinação dos fatores com significância estatística e com significância prática. Foram realizadas comparações múltiplas de médias segundo o teste de Duncan ou LSD de Fisher para verificar entre quais perfis realmente existiam diferenças. O objetivo dessa análise foi comparar médias entre três ou mais grupos em relação a uma variável de nível intervalar (contínua ou discreta) de interesse para identificar possíveis níveis de criticidade diferentes. Os pressupostos para a utilização desta análise, a normalidade dos resíduos do modelo e a presença de variância constantes, foram verificados.

O projeto foi aprovado no Comitê de Ética em Pesquisa da Faculdade de Medicina da Universidade Federal de Minas Gerais, sob o número 34133814.5.0000.5149. Foi obtida a dispensa do termo de consentimento livre e esclarecido.

## RESULTADOS

Observou-se maior tempo de permanência hospitalar de mulheres pertencentes aos grupos etários de 15 a 17 anos e 45 anos ou mais quando comparados com os demais grupos (18 a 24 anos, 25 a 34 anos e 35 a 44 anos), com diferenças estatisticamente significativas ( Tabela 1 ).

**Tabela 1:** Tempo de permanência hospitalar de mulheres submetidas à cesárea em relação ao grupo etário das categorias diagnósticas relacionadas ao MDC14. Brasil, 2012–2017.

Variável	Permanência hospitalar após a cesariana (dias)		

	n (%)	Média (DP)	p
Grupo etário (anos)			**0,050**
15 a 17^a^	632 (1,0)	2,5 (1,5)	
18 a 24^b^	8004 (12,4)	2,3 (1,4)	
25 a 34^b^	37498 (58,1)	2,2 (1,4)	
35 a 44^b^	18100 (28,1)	2,3 (1,4)	
45 ou mais^a^	239 (0,4)	2,6 (1,7)	

p-valor em negrito < 0,05 na Anova fatorial.

Letras iguais significam similaridade entre as médias dos grupos.

Em relação aos CID, de um total de 81 avaliados, foi identificada influência significativa de 63 deles. No entanto, 34 desses CID apresentaram efeito pequeno (d de Cohen < 0,40) – ou seja, apesar de existir significância estatística, não houve significância prática. Por isso, foram avaliados individualmente 29 CID, sendo que 12 mostraram uma relevância maior (d de Cohen ≥ 0,60) e os demais uma relevância moderada (d de Cohen entre 0,40 e 0,60), conforme demonstrado na Tabela 2 . Observa-se que o tempo de permanência hospitalar de mulheres submetidas à cesariana com complicações ou comorbidades foi maior que o das mulheres submetidas à cesariana sem complicações ou comorbidades para todos os CID.

**Tabela 2:** Tempo de permanência hospitalar de mulheres submetidas à cesárea em relação às complicações ou comorbidades presentes à admissão segundo o CID das condições relacionadas ao MDC14. Brasil, 2012–2017.

Variável		Permanência hospitalar após cesariana (dias)			

		Comorbidades secundárias		Anova	d de Cohen
	
CID	Descrição do CID	Ausentes Média (DP)	Presentes Média (DP)		
O15	Eclâmpsia	2,3 (1,4)	4,5 (2,2)	< 0,001	1,590
O11	Distúrbio hipertensivo pré-existente com proteinúria superposta	2,3 (1,4)	4,4 (2,0)	< 0,001	1,503
O14	Hipertensão gestacional (induzida pela gravidez) com proteinúria significativa	2,2 (1,4)	3,8 (2,0)	< 0,001	1,109
R60	Edema não classificado em outra parte	2,3 (1,4)	3,7 (2,1)	0,008	1,035
E14	Diabetes *mellitus* não especificado	2,3 (1,4)	3,5 (1,8)	< 0,001	0,840
O12	Edema e proteinúria gestacionais (induzidos pela gravidez), sem hipertensão	2,3 (1,4)	3,4 (1,6)	< 0,001	0,790
D69	Púrpura e outras afecções hemorrágicas	2,3 (1,4)	3,2 (1,7)	0,010	0,686
D64	Outras anemias	2,3 (1,4)	3,2 (2,0)	< 0,001	0,683
O26	Assistência materna por outras complicações ligadas predominantemente à gravidez	2,3 (1,4)	3,2 (1,7)	< 0,001	0,619
E10	Diabetes *mellitus* insulino-dependente	2,3 (1,4)	3,1 (2,1)	0,004	0,585
O23	Infecções do trato geniturinário na gravidez	2,3 (1,4)	3,1 (1,9)	< 0,001	0,577
O43	Transtornos da placenta	2,3 (1,4)	3,1 (2,0)	< 0,001	0,571
O30	Gestação múltipla	2,3 (1,4)	3,0 (1,8)	< 0,001	0,537
O44	Placenta prévia	2,3 (1,4)	3,0 (1,8)	< 0,001	0,527
O60	Trabalho de parto pré-termo	2,3 (1,4)	3,0 (1,8)	< 0,001	0,519
O84	Parto múltiplo	2,3 (1,4)	3,0 (1,7)	< 0,001	0,497

CID: Classificação Internacional de Doenças

Ao combinar o grupo etário e os 16 CID com relevância estatística e prática, foram identificados quatro níveis de criticidade entre mulheres submetidas à cesariana. O nível de criticidade 1 foi composto por mulheres submetidas à cesariana, independentemente do grupo etário e sem a ocorrência de complicações ou comorbidades presentes à admissão. O nível de criticidade 2 foi formado por mulheres de qualquer grupo etário submetidas à cesariana e com apenas uma complicação ou comorbidade presente à admissão, que não fosse dos CID O15, O11, O14, E14 e O12, ou a combinação dos CID O60 e 84 ( Quadro ). O nível de criticidade 3 foi constituído por mulheres do grupo etário de 18 a 44 anos que possuíam uma combinação de duas ou três complicações ou comorbidades presentes à admissão ou apenas os CID O11, O12, O14 e E14. O nível de criticidade 4 foi composto por mulheres pertencentes ao grupo etário de 15 a 17 anos e 45 anos ou mais, que apresentavam uma combinação de duas ou três complicações ou comorbidades presentes à admissão ou apenas os CID O11, O12, O14 e E14. Havia, também, mulheres de qualquer grupo etário que apresentavam CID O14 (hipertensão gestacional induzida pela gravidez com proteinúria significativa) combinado com qualquer outro CID com relevância estatística e prática que não fosse CID O15 (eclâmpsia). Mulheres que possuíam esse diagnóstico de eclâmpsia foram categorizadas, independentemente do grupo etário, no nível de criticidade 4 ( Quadro ).

**Quadro:** Níveis de criticidade para permanência hospitalar de mulheres submetidas à cesariana das categorias diagnósticas relacionadas à MDC14. Brasil, 2012–2017.

Nível de criticidade	Grupo etário (anos)	CID															

		O15	O11	O14	R60	E14	O12	D69	D64	O26	E10	O23	O43	O30	O44	O60	O84
1	Todos	Ausência de complicações ou comorbidades presentes à admissão															
2	Todos	Presença de pelo menos uma complicação ou comorbidade presente à admissão (CID O15, O11 e O14)															
3	18 a 44													Sim			Sim
	18 a 44													Sim		Sim	
	18 a 44													Sim		Sim	Sim
	18 a 44						Sim										
	18 a 44					Sim											
	18 a 44			Sim													
	18 a 44			Sim												Sim	
	18 a 44			Sim										Sim			
	18 a 44			Sim								Sim					
	18 a 44		Sim														
4	≤ 17 / ≥ 45													Sim			Sim
	≤ 17 / ≥ 45													Sim		Sim	
	≤ 17 / ≥ 45													Sim		Sim	Sim
	≤ 17 / ≥ 45						Sim										
	≤ 17 / ≥ 45					Sim											
	≤ 17 / ≥ 45			Sim													
	≤ 17 / ≥ 45			Sim												Sim	
	≤ 17 / ≥ 45			Sim										Sim			
	≤ 17 / ≥ 45			Sim								Sim					
	≤ 17 / ≥ 45		Sim														
	Todos											Sim				Sim	
	Todos			Sim													Sim
	Todos			Sim						Sim							
	Todos			Sim					Sim								
	Todos			Sim				Sim									
	Todos			Sim			Sim										
	Todos			Sim	Sim												
	Todos	Sim															

CID: Classificação Internacional de Doenças

Por fim, na Tabela 3 , verifica-se o tempo de permanência hospitalar segundo o nível de criticidade dos grupos. Observa-se que a média do tempo de permanência aumenta junto com o nível de criticidade.

**Tabela 3:** Tempo de permanência hospitalar de mulheres submetidas à cesárea em relação aos níveis de criticidade das categorias diagnósticas relacionadas à MDC14. Brasil, 2012–2017.

Variável	Permanência hospitalar após cesariana (dias)					

	Média (DP)	p10	p25	p50	p75	p90
Nível de criticidade						
1	2,2 (1,3)	1,7	1,9	2,1	2,4	3,0
2	2,7 (1,7)	1,8	2,0	2,3	3,1	5,1
3	3,6 (1,9)	1,9	2,2	3,0	5,1	9,5
4	4,5 (2,0)	2,1	2,6	4,4	6,9	10,7

## DISCUSSÃO

Este estudo demonstrou maior tempo de permanência hospitalar após cesárea em mulheres jovens (15 a 17 anos) e com idade mais avançada (45 anos ou mais) em relação às demais faixas etárias. Foi revelado ainda que as comorbidades associadas à gestação que mais aumentam o número de dias de internação são a eclâmpsia, o distúrbio hipertensivo pré-existente com proteinúria superposta e a hipertensão gestacional (induzida pela gravidez) com proteinúria significativa, entre outras. Além disso, o presente trabalho possibilitou a construção de perfis distintos de níveis de criticidade a partir da combinação de grupos etários e das principais comorbidades, os quais se apresentaram diretamente relacionados ao tempo de permanência hospitalar.

Estudo realizado em 30 países de baixa e média renda demonstrou que a média de internação após cesariana variou entre 2,5 e 9,3 dias nas localidades pesquisadas. Conforme os achados do presente estudo, um dos fatores associados ao maior tempo de permanência após o procedimento foi a idade materna avançada, assim como em estudo realizado nos Estados Unidos, no qual mais dias de internação foram associados a extremos de idade (< 18 ou > 35 anos) e a múltiplas comorbidades gestacionais^12^ . Segundo os autores, conhecer os fatores de risco associados aos períodos prolongados de internação possibilita a realização de ações de monitoramento e vigilância durante a gestação, sobretudo na presença de mais de uma patologia^13^ .

No Brasil, o número de mulheres com gestações tardias tem aumentado nos últimos anos^14^ . Nota-se que isso ocorre por razões diversas, tais como maior dedicação ao estudo e profissão, alta disponibilidade de métodos contraceptivos, além de casamentos tardios e escolha de parceiros ideais^15^ . Contudo, a elevada faixa etária materna frequentemente associa-se a riscos gestacionais. Estudo de coorte retrospectiva realizada no Texas, com 96.879 participantes, demonstrou maiores taxas de pré-eclâmpsia, eclâmpsia e placenta prévia em gestantes de 40 anos ou mais do que naquelas de 35 a 39 anos^15^ . Assim como neste estudo, tais mulheres enquadram-se em níveis de criticidade superiores, os quais devem ser considerados no planejamento da permanência hospitalar pós-parto e do cuidado, proporcionando melhores desfechos perinatais.

Os resultados desta pesquisa evidenciaram que os distúrbios hipertensivos na gestação são as comorbidades mais associadas a períodos prolongados de internação após cesárea. Esses dados são confirmados por estudos anteriores, nos quais a presença dessas complicações associou-se a maiores taxas de cesarianas, prolongados períodos de internação e readmissão hospitalar, além de elevados custos para o sistema de saúde^15 , 16^ . Dessa forma, enfatiza-se a importância das ações de prevenção e promoção da saúde durante o pré-natal, a fim de minimizar a carga de morbidade e mortalidade materna populacional.

O presente estudo possibilitou ainda a estratificação das mulheres em quatro diferentes níveis de criticidade e demonstrou a necessidade de prolongamento do tempo de internação a partir da associação entre o somatório das comorbidades na gestação e a idade materna, mediante necessidades clínicas diversas. O reconhecimento dos diferentes riscos obstétricos e dos níveis de criticidade de mulheres submetidas à cesariana auxilia na proposição de estratégias que minimizem o tempo de internação hospitalar e subsidiem a tomada de decisão na prática clínica. Nesse sentido, o presente estudo ampara a criação de pontos de corte baseados na estratificação de risco dessas mulheres a fim de que elas recebam assistência adequada e segura a partir de uma estimativa do tempo esperado de hospitalização.

Cita-se como limitação destes achados a não representatividade da amostra, uma vez que os hospitais foram escolhidos de forma não aleatória. Foi utilizado o banco de dados disponível para os pesquisadores, uma amostra de conveniência que pode não representar a totalidade da rede hospitalar brasileira. Contudo, cabe destacar a potencialidade dos referenciais de risco aqui apresentados para subsidiar a atuação dos gestores e profissionais de saúde no sentido de planejar a assistência a gestantes e puérperas em todo o país.

O sistema DRG está associado à redução do tempo de internação após cesariana e de outros procedimentos obstétricos e ginecológicos, além de reduzir as taxas de partos cirúrgicos^17^ . Essa metodologia possibilita a otimização do uso de recursos hospitalares aliada à melhoria da qualidade dos atendimentos e da segurança dos pacientes, impactando diretamente na redução das taxas de morbidade e mortalidade materna^18^ .

## CONSIDERAÇÕES FINAIS

O estudo mostrou que o DRG é uma ferramenta que influencia na gestão de custos e de cuidados. Por meio dela, foi possível a criação de níveis de criticidade, o que possibilitou prever o tempo médio de permanência hospitalar de mulheres submetidas à cesariana. Caso o tempo de permanência fosse maior do que o calculado pelo método, seria indicativo de uma falha no processo assistencial que, consequentemente, poderia gerar aumento dos custos hospitalares e ônus ao paciente.

A avaliação do impacto das condições clínicas e do grupo etário no tempo de permanência hospitalar das mulheres submetidas à cesariana é, indubitavelmente, fator a ser considerado ao se pensar em qualidade e eficiência da assistência. Este estudo é, portanto, de importância para o planejamento do cuidado e da gestão hospitalar.
